# Iron Metabolism of the Skin: Recycling versus Release

**DOI:** 10.3390/metabo13091005

**Published:** 2023-09-12

**Authors:** Marta Surbek, Supawadee Sukseree, Leopold Eckhart

**Affiliations:** Department of Dermatology, Medical University of Vienna, 1090 Vienna, Austria; marta.surbek@meduniwien.ac.at (M.S.); supawadee.sukseree@meduniwien.ac.at (S.S.)

**Keywords:** epidermis, epithelium, ferroptosis, ferroportin, heme oxygenase, hair, hephaestin, keratinocytes, microbiome, oxidative stress

## Abstract

The skin protects the body against exogenous stressors. Its function is partially achieved by the permanent regeneration of the epidermis, which requires high metabolic activity and the shedding of superficial cells, leading to the loss of metabolites. Iron is involved in a plethora of important epidermal processes, including cellular respiration and detoxification of xenobiotics. Likewise, microorganisms on the surface of the skin depend on iron, which is supplied by the turnover of epithelial cells. Here, we review the metabolism of iron in the skin with a particular focus on the fate of iron in epidermal keratinocytes. The iron metabolism of the epidermis is controlled by genes that are differentially expressed in the inner and outer layers of the epidermis, establishing a system that supports the recycling of iron and counteracts the release of iron from the skin surface. Heme oxygenase-1 (HMOX1), ferroportin (SLC40A1) and hephaestin-like 1 (HEPHL1) are constitutively expressed in terminally differentiated keratinocytes and allow the recycling of iron from heme prior to the cornification of keratinocytes. We discuss the evidence for changes in the epidermal iron metabolism in diseases and explore promising topics of future studies of iron-dependent processes in the skin.

## 1. Metabolism of Iron in the Skin

### 1.1. Functions of Iron in Skin Cells

The function of skin as an organ of protection depends largely on its epithelial compartment, the epidermis, which is in direct contact with the environment. Skin appendages, such as hair, nails, and sweat glands, are specialized extensions of the epithelium, with contributions from the mesenchyme mainly during their development and regeneration [1]. The epidermis of the skin is a prototypical stratified epithelium in which cells proliferate in the basal layer, at the interface to the underlying dermis, and subsequently move towards the surface, thereby forming suprabasal cell layers. The changes in gene expression and cellular structure during this movement are known as epithelial cell differentiation [2,3,4]. Ultimately, the cells are shed from the surface so that the total cell number of the epithelium is maintained as a constant under the conditions of tissue homeostasis. Disturbances of the tissue by exogenous stressors or endogenous inflammation typically lead to hyperproliferation and modifications of differentiation, resulting in epithelial thickening. The homeostasis and dynamic changes of the epidermis depend on the tight regulation of the cellular metabolism. The present review focuses on the metabolism of iron, a long-standing topic that has recently seen substantial progress because of new insights from studies in mouse and cell culture models. Many steps of the iron turnover are conserved in all cell types and have been summarized in recent excellent review articles [5,6,7]. However, there are several specific features, such as the regulated expression of regulators of iron metabolism and transport, that point to unique adaptations of the iron metabolism in the epidermis [8,9,10].

Iron is required for central cellular processes, such as mitochondrial respiration, DNA synthesis and detoxification reactions, which are also essential for epithelia. In most of its physiological roles, iron is coordinated, in the form of the ferrous cation (Fe^2+^), with protoporphyrin IX, which is composed of four modified pyrrole subunits interconnected by methine bridges. This coordination complex, heme, is very stable and acts as a prosthetic group of many proteins, collectively known as hemoproteins. In these proteins, iron is also coordinated with a histidine residue. The most well-known hemoprotein is hemoglobin, which carries oxygen. Other hemoproteins such as cytochrome c are involved in electron transfer within the electron transport chain and are therefore essential for oxidative phosphorylation in mitochondria. Yet, other hemoproteins, such as cytochrome P450s, catalases, and peroxidases, have catalytic activity and use oxygen for oxidation or hydroxylation reactions. Besides hemoproteins, iron–sulfur proteins are the second main class of iron metalloproteins. Examples are succinate–coenzyme Q reductase (part of complex II of the electron transport chain), coenzyme Q–cytochrome c reductase (part of complex III of the electron transport chain), ferredoxins, and NADH dehydrogenase. Aconitase is an iron–sulfur protein that catalyzes the isomerization of citrate in the tricarboxylic acid cycle. Mononuclear iron dioxygenases, such as lipoxygenases, utilize dioxygen (O_2_) for the oxidation of their substrate. Ribonucleotide reductase, belonging to the class of oxo-bridged diiron proteins [11], catalyzes the conversion of ribonucleotides into deoxyribonucleotides and thereby facilitates DNA synthesis. Iron is stored intracellularly in a complex with ferritin, which is another oxo-bridged diiron protein [12], and transported through the blood plasma via transferrin, a glycoprotein with two binding sites for ferric iron ions (Fe^3+^) [13].

Although iron is essential for many processes, free iron ions, especially Fe^2+^, can have detrimental effects on cells. The oxidation reaction between Fe^2+^ and H_2_O_2_, known as the Fenton reaction, generates hydroxyl radicals, which react with fatty acids to form lipid radicals which further initiate lipid peroxidation. Together with lipoxygenase-dependent lipid peroxidation, the Fenton reaction can lead to a specific type of programmed cell death called ferroptosis [14,15,16]. The downregulation of lipoxygenase activity can protect cells from iron-induced programmed cell death [17]. It has been suggested that lipid peroxidation contributes to psoriasis [18] but also to normal epidermal differentiation and epidermal barrier formation [19,20]. Suppressors of lipid peroxidation were found to be upregulated in the basal epidermal layer, while arachidonate lipoxygenase 12 (ALOX12) and a product of lipid peroxide decomposition, 4-hydroxynonenal, were found to be increased in the uppermost granular layer [20]. The importance of lipid peroxidation in the terminal differentiation of keratinocytes is supported by the association of ALOX12B and ALOXE3 loss-of-function mutations with non-bullous congenital ichthyosiform erythroderma, a keratinocyte cornification disorder [21]. However, there is no substantial evidence supporting speculations about ferroptosis as a mechanism of keratinocyte differentiation-associated cell death.

### 1.2. Roles of the Epidermis in the Regulation of Systemic Iron Levels

The iron balance of the organism is maintained by the uptake, storage, and removal/loss of iron. The skin is involved in the latter two processes, whereas the organismal acquisition of iron occurs exclusively through iron absorption in the gastrointestinal tract. The role of the skin as an iron depository is documented in humans and mice. The skin of thalassemia patients with iron overload contains 10 times more iron than healthy skin [22]. Consistent with this, the epidermal iron content increased 100-fold upon a single intraperitoneal injection of iron dextran in a mouse model of iron overload [23]. Therefore, regulation of iron homeostasis and recycling in the skin is required not only for proper functioning of the keratinocytes, but also for maintaining systemic iron levels.

The excretion or loss of iron occurs through the desquamation of keratinocytes, besides loss through enterocytes, menstruation, and childbirth [13]. Normal iron loss from all sources reaches 1 mg/day, of which a great part is lost through the skin [24]. An epidermal function for systemic iron regulation has been further demonstrated in a model of increased iron import in keratinocytes. Upon increased epidermal iron import and increased desquamation, systemic iron levels were reduced [25]. Furthermore, it is estimated that up to 2.5 mg/day of iron can be lost in conditions associated with enhanced desquamation, such as psoriasis [26]. Moreover, it has been reported that people diagnosed with psoriasis tend to be deficient in iron and display markers of depleted iron stores [27]. This could be explained in part by the extensive exfoliation of skin squames containing high levels of iron [23]; however, the etiology of iron deficiency in exfoliative skin diseases is yet to be confirmed.

The iron concentration in healthy human skin is approximately 0.15–0.275 mg/g [22,28]. However, the iron content of the epidermis is approximately 10 times less, with a mean value determined in one study to be 26 µg/g [29]. The iron concentration in the epidermis varies between different layers, depending on the differentiation stage of epidermal keratinocytes. A previous study investigated the abundance of iron over skin cross sections and found the highest iron concentration in the basal layer and a consistently lower concentration in the stratum corneum [30]. This study also detected elevations of iron in the epidermis of patients with psoriasis, a result that is consistent with an earlier report [29], and atopic dermatitis. Specifically, Forslind and colleagues reported the following iron concentrations in healthy and diseased skin (mean ± SD, ng/mg, values are rounded here): 106 ± 85 (basal, normal), 17 ± 27 (stratum corneum, normal), 141 ± 87 (basal, psoriasis), 36 ± 45 (stratum corneum, psoriasis), 170 ± 106 (basal, atopic dermatitis), and 82 ± 42 (stratum corneum, atopic dermatitis) [30]. In a rat model of skin wound healing, the iron concentration in the skin increased to more than four-fold the level of normal skin with a peak on the third day after wounding, thus coinciding with the peak of cell proliferation [28].

## 2. Molecular Control of Iron Metabolism in Epithelial Cells

### 2.1. Iron Import in the Basal Layer

Iron is absorbed from the gastrointestinal tract and transported to tissues in the form of Fe^3+^ complexed with transferrin. Basal epidermal keratinocytes express transferrin receptor 1 (TFR1, encoded by the gene *TFRC*, also known as CD71) [31], to which the transferrin–Fe^3+^ complex is bound. The resulting complex is internalized via endocytosis through clathrin-coated pits. Basal keratinocytes can be divided into CD71^high^ (keratinocyte stem cells) and CD71^low^ (transitory amplifying cells)-expressing cells, suggesting that proliferating basal keratinocytes rapidly lose CD71 during early differentiation [32]. TFR1-independent iron transportation systems have been described in the heart and liver, but to the best of our knowledge, there is solid evidence only for TFR1-dependent iron transport in the skin. Importantly, TFR1 is one of the key control mechanisms that regulate intracellular iron concentrations. TFR1 expression is induced in iron-deprived conditions to enhance iron uptake [23]. The overexpression of TFR1 suffices to increase the intracellular iron storage in proliferating and differentiated keratinocytes [33]. In non-epithelial cells of the skin, TFR1 expression is upregulated in malignantly transformed melanocytes, as well as in high-grade malignancy cutaneous lymphomas, in comparison to benign melanocytic nevi or low-grade lymphomas [31]. Thus, the expression level of TFR1 plays a central role in balancing intracellular iron levels of keratinocytes and other skin cells.

**Figure 1:**
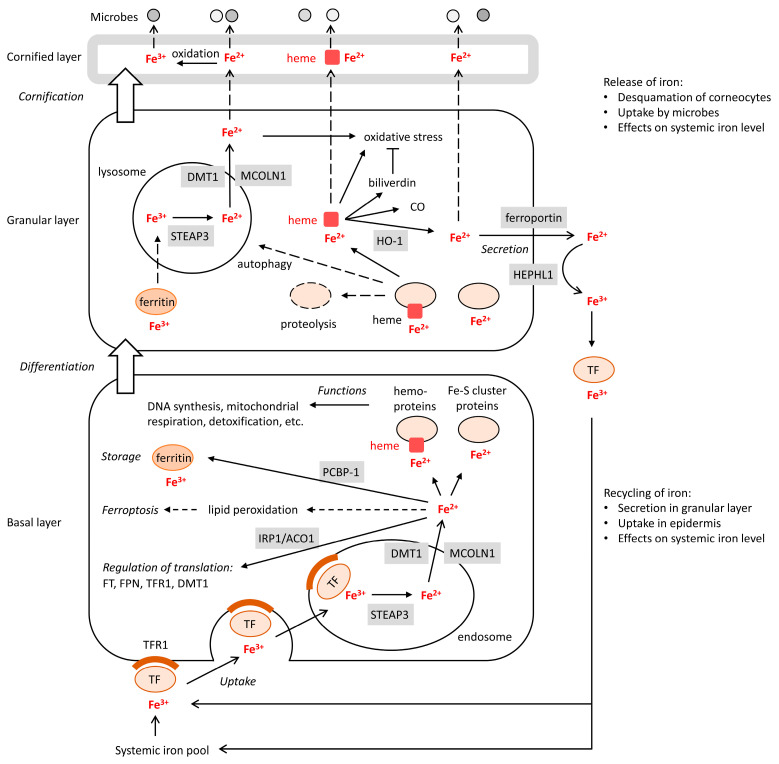
Schematic depiction of the iron metabolism in the epidermis. The process of keratinocyte differentiation is represented by cells in the basal layer, the granular layer and the cornified layer. The distribution of iron-binding proteins and regulators of iron metabolism and transport is shown according to the evidence and hypotheses discussed in the main text. Alternative pathways of iron turnover lead either to the release of iron from the epidermal surface, associated with the uptake of a fraction of total iron by microbes, or to the recycling of iron. Details are described in the main text. DMT1, divalent metal transporter 1; FT, ferritin; FPN, ferrportin; HEPHL1; hephaestin-like 1; IRP1, iron regulatory protein 1; MCOLN1, mucolipin TRP cation channel 1; PCBP1, poly(rC) binding protein 1; STEAP3, six-transmembrane epithelial antigen of prostate 3; TF, transferrin; TFR1, transferrin receptor 1.

In the internalized complex, transferrin–TFR1–Fe^3+^ localizes to endosomes. As the binding of TFR1 to Fe^3+^ strongly decreases at lower pH, the acidification of the environment causes the release of Fe^3+^ from TFR1. Subsequently, Fe^3+^ is reduced to Fe^2+^ by STEAP3 (six-transmembrane epithelial antigen of prostate 3) in the endosome. Fe^2+^ is translocated into the cytoplasm via DMT1 (divalent metal transporter 1, gene name: *SLC11A2*) or mucolipin TRP cation channel 1 (MCOLN1) (Figure 1).

### 2.2. Iron Storage in the Epidermal Keratinocytes

In the cytoplasm, iron is stored in a complex with ferritin, which consists of 24 subunits that can bind up to 4500 iron atoms. The outer shell of this complex is built by ferritin heavy chain (FTH) and ferritin light chain (FTL) proteins. Of note, FTH has ferroxidase activity, allowing it to oxidize Fe^2+^. The sequestration of iron by ferritin is important in order to prevent its toxic effects such as lipid peroxidation. Ferritin is expressed in both the dermis and epidermis of human skin [34]; however, epidermal ferritin levels are 2–7-fold higher [35]. In the epidermis, ferritin is most abundant in the basal layer, according to two previous studies [9,34], although a recent paper detected ferritin by immunostaining in all epidermal layers [23]. The expression of ferritin is regulated on the transcriptional, translational, and post-translational levels. The transcription factor Nrf2, critically controlling the antioxidant responses of most cells, upregulates the mRNA of FTH and FTL [36]. The translation of these mRNAs is regulated by IRPs depending on the levels of free iron ions, as described in Section 2.3 [37]. Finally, the transfer of ferritin to lysosomes through NCOA4-dependent ferritinophagy [38] leads to its breakdown and the release of iron ions (Figure 1).

The expression of ferritin is increased in an Nrf2-dependent manner upon ultraviolet A (UVA) irradiation, whereby free iron ions are required to support the efficient production of ferritin proteins [39]. Free Fe^2+^ also causes lipid peroxidation, which can be prevented by the chelation of free iron [34]. The overexpression of FTH in transgenic mice caused epidermal hyperplasia with hyperkeratosis and hair loss [40]. The underlying mechanisms are not known but may include iron-independent processes.

### 2.3. Regulation of Iron Homeostasis by Iron Regulatory Proteins

To support the utilization of iron and reduce the potentially detrimental effects of iron, the equilibrium between free Fe^2+^ and sequestrated Fe^3+^ is subjected to tight regulation in epidermal keratinocytes as well as in other cells. The regulation of iron homeostasis depends largely on the concentration of free Fe^2+^ in the cytoplasm. Fe^2+^ binds to two iron regulatory proteins (IRPs), iron regulatory protein 1 (IRP1), which has a second function as an enzyme, aconitase, and iron regulatory protein 2 (IRP2). When iron is deficient, IRPs bind to iron regulatory element (IRE) in the 5′ or 3′ untranslated regions (UTRs) of mRNAs of iron-binding proteins. The binding of IRPs to the 5′ UTR of ferritin heavy and light chain (FTH and FTL) and ferroportin mRNAs prevents their translation, whereas the binding of IRPs to the 3′ UTR of TFR1 mRNA stabilizes the mRNA and thereby increases the rate of TFR1 translation. In this manner, the levels of proteins involved in iron storage, i.e., ferritin, and iron export, i.e., ferroportin, decrease when cytoplasmic free Fe^2+^ is low, while the formation of the iron-import-related protein TFR1 is increased [41]. In human epidermis both IRP1 and IRP2 are expressed predominantly in the basal layer [9]. UVA irradiation leads to a loss of the binding capacity of IRPs to IRE and consequently to a disbalance of the cellular iron equilibrium [42].

While intracellular iron turnover is controlled by IRPs, extracellular iron turnover is controlled to a large extent by hepicidin, an iron regulatory hormone encoded by the gene *HAMP*. Hepcidin regulates the iron levels of plasma by inducing the degradation of ferroportin and thereby the transfer of iron into the plasma from ferroportin-positive cells. Although hepcidin is mainly produced in the liver, other organs such as the skin also produce hepcidin, which may play tissue-specific roles. In the skin, hepcidin is not detectable under normal conditions, but its expression in epidermal keratinocytes is induced by infection with group a Streptococcus (GAS) [43]. The genetic deletion of hepcidin specifically in keratinocytes rendered mice highly sensitive to the systemic spread of GAS in a model of necrotizing fasciitis. The protective effect of inducible keratinocyte-derived hepcidin was attributed to the induction of the chemokine CXCL1, whereas the concentrations of iron in the skin and plasma were not significantly altered in this mouse model [43].

## 3. Recycling and Transport of Iron in Differentiated Epidermal Keratinocytes

### 3.1. Metabolism of Hemoproteins, Heme, and Iron Ions in Differentiated Keratinocytes

The skin serves as a large depository of systemic iron. The basal layer of the epidermis contains significantly more iron than the upper layers, thereby establishing an iron gradient [30]. Due to the constant desquamation of the epidermis, iron losses are huge, despite the gradient of iron concentration in the epidermis that minimizes its loss. The gradient of iron concentration between layers is enabled by the specific iron metabolism in the upper layers, which is mostly directed towards iron recycling. Proteins involved in iron import and storing, such as transferrin receptor 1 (TFR1), DMT1, IRP1 and IRP2, and FT, are highly expressed in the basal layer, whereas proteins mediating the release of iron from heme and the export of iron, namely heme oxygenase 1 (HO-1) and ferroportin, respectively, are upregulated in the granular layer of the epidermis [9] (Figure 1). The theory that iron is recycled in the upper layers is supported by the finding that only approximately 10% of iron that is incorporated into the epidermis is eventually lost [44].

Iron is excreted from the epidermis via the stratum corneum, the outermost layer of the skin. The stratum corneum consists of corneocytes, i.e., dead keratinocytes that are largely filled with cross-linked proteins [3,45], and extracellular material, which largely consists of lipids. The corneocytes and the material surrounding them are released to the environment by desquamation under the control of proteases that cleave intercellular connections (desmosomes). Iron can enter the stratum corneum either as a component of keratinocytes that undergo cornification and thereby leave the granular layer or through the intercellular space. However, the latter route is controlled by tight junctions, located in the granular layer of the epidermis in healthy skin [46]. Tight junctions block the passage of proteins and thereby also prevent the passage of iron, which is efficiently bound by proteins. Accordingly, the epidermal excretion rate of iron can be increased in three ways: (1) an increase in the amount of iron in keratinocytes at the time of cornification, (2) an increase in the rate of keratinocytes undergoing cornification, e.g., in hyperproliferative skin diseases, and (3) the impairment of the barrier function of tight junctions.

The amount of iron in cornifying keratinocytes is probably constant during the passive movement of the cells from the basal layer to the granular layer. This assumption is based on the low abundance of proteins involved in the uptake or secretion of iron in the middle layers of the epidermis [9]. Experimental studies are needed to substantiate this point. However, iron turnover appears to be activated in the granular layer (see below for details) such that the intracellular binding of iron to proteins is altered and a fraction of the iron ions are secreted. This decrease in the iron content of granular layer cells implies a reduction in the iron concentration in the stratum corneum as compared to the lower layers of the epidermis. Conversely, if the molecular control of iron secretion is impaired, the intracellular concentration of iron within the cells of the granular and cornified layers will increase. Such a disturbance of the normal processes of the granular layer occurs in many diseases that are characterized by altered and accelerated keratinocyte differentiation, such as psoriasis. The accelerated production of corneocytes, macroscopically visible as scales, increases the loss of metabolites per area of the skin, even if the turnover of molecules in the granular layer would not be altered. The third theoretical mechanism of enhanced iron excretion involves defects of tight junctions. Although such defects have been reported in atopic dermatitis [47] and the mislocalization of tight junctions in lower layers of the epidermis has been determined in psoriasis [48], the consequences on the localization of iron-binding proteins in these diseases has not been studied in detail. Notably, a proteomic analysis found that the amount of the extracellular iron carrier transferrin (encoded by the gene *TF*), also referred to as serotransferrin, was strongly elevated (12-fold, paired effect lesional vs. nonlesional, *p* = 0.00004, *n* = 24) and hemopexin (encoded by the gene *HPX*), the main protein binding heme in the extracellular space, was also elevated (two-fold, *p* = 0.03) in the same study [49].

### 3.2. Expression and Activity of Heme Oxygenase 1 (HO-1) in Differentiated Keratinocytes

*HMOX1* (heme oxygenase 1, HO-1) is one of three main iron-metabolism-related genes, besides *SLC40A1* (ferroportin) and *HEPHL1* (hephaestin-like 1) (see below), which are transcriptionally upregulated in differentiated keratinocytes. HO-1 catalyzes the degradation of heme into biliverdin, carbon monoxide (CO) and Fe^2+^. The expression of *HMOX1* is induced by Nrf2 in many cell types, including proliferating keratinocytes, under conditions of oxidative stress, and Nrf2-dependent HO-1 expression has also been reported for the inflammatory skin disease psoriasis [50,51,52]. In contrast to the inducible expression of *HMOX1*, differentiated keratinocytes of the upper epidermis contain constitutively high levels of HO-1 [9,10,53]. Both mRNA and protein levels of HO-1 increase during the differentiation of epidermal keratinocytes in vitro [10]. Likewise, the transcriptional activity is induced by the *HMOX1* promoter and the HO-1 protein is present in the suprabasal epidermis of unstressed skin [9,10,54]. The regulation of *HMOX1* gene expression remains to be investigated, for example, by comparing wildtype and Nrf2 knockout mice.

To exert its enzymatic activity, HO-1 depends on its substrate, heme. It was proposed that heme is released from hemoproteins in the course of the intracellular remodeling prior to cornification [10]. Multiple hemoproteins, such as CYP450 protein and cytochrome c, are present in epidermal keratinocytes. In the final steps of keratinocyte differentiation, proteases degrade non-cytoskeletal proteins of the cytosol and organelles, so that heme can be set free. However, this plausible model has not yet been tested experimentally. The immunolabeling of cytochrome c demonstrated a change from a granular to a homogenous cytoplasmic distribution in the upper granular layers and a loss of immunoreactivity in the stratum corneum [55], which is in line with the degradation of cytochrome c during cornification.

The products of HO-1, biliverdin and CO, were reported to exert antioxidant and anti-inflammatory activities, respectively, in other tissues. They may have similar roles in the granular layer of the epidermis, but these remain to be investigated. Importantly, ferrous iron ions are released from heme. It is presently not known whether Fe^2+^ causes lipid peroxidation or other pro-oxidant effects in the granular layer, but the co-expression of ferroportin in these cells suggests that they are exported from the cell. Deletion of *Hmox1* in keratinocytes (*Hmox1^f/f^ K14-Cre*) did not lead to gross alterations in the skin or skin appendages of mice [10]. However, the potential impact of this gene deletion on the resistance to oxidative stress [56,57] and iron levels in the stratum corneum and indirect effects on the skin microbiome were not investigated in a recently published study [10].

### 3.3. Expression and Function of Ferroportin (SLC40A1) in Differentiated Keratinocytes

Ferroportin (FPN, SLC40A1) is an iron exporter that is expressed at high levels in the granular layer of the epidermis [9]. Ferroportin transports Fe^2+^ ions from the cytoplasm into the extracellular space [58]. The suppression of ferroportin expression using siRNAs led to a small but significant increase in the iron content of human keratinocytes differentiating in vitro [9]. The deletion of ferroportin specifically in the keratinocytes (*Slc40a1^f/f^ K14-Cre*) of mice led to an approximately two-fold elevation of the iron content in the stratum corneum. These mice did not develop an obvious skin phenotype, at least under standard housing conditions. When fed a low-iron diet, the epidermal ferroportin knockout mice suffered from systemic effects, such as reduced hemoglobin content in the blood, possibly caused by the decreased recycling of iron from the upper epidermis, and a lower body weight [9]. The study by Asano and colleagues [9] provided valuable insights into the epidermal iron metabolism, as it demonstrated that (1) the iron turnover in the granular layer, i.e., the site of ferroportin expression in normal mice, controls the iron content of the stratum corneum, and (2) the metabolism of iron in the epidermis can affect systemic iron levels.

### 3.4. Expression and Function of Hephaestin-like 1 (HEPHL1) in Differentiated Keratinocytes

The evidence for the ferroportin-mediated secretion of iron from keratinocytes of the granular layer raises the question of the further fate of the secreted iron ions. Notably, ferroportin secretes Fe^2+^, which is potentially toxic and has to be oxidized to Fe^3+^ to allow binding to the extracellular iron transporter transferrin. Upon dietary absorption of iron in enterocytes, Fe^2+^ ions are also secreted by ferroportin into the plasma, where they are oxidized in a dioxygen (O_2_)-dependent manner by hephaestin (encoded by the gene *HEPH*). Hephaestin contains a domain homologous to ceruloplasmin, the ferroxidase of blood, and a transmembrane domain that anchors the protein to the outer surface of the cell membrane [59]. HEPH is predominantly expressed in the duodenum, intestine, and colon and the expression of HEPH in the epidermis was limited to the basal layer in an immunostaining study [9]. However, a structurally similar paralog, hephaestin-like 1, encoded by the gene *HEPHL1* and also referred to as zyklopen, is predominantly expressed in the esophagus, skin, placenta, thyroid and testis. To the best of our knowledge, an immunolocalization study of HEPHL1 has not yet been published, but a series of published data indicate the expression of HEPHL1 in the suprabasal layers of the epidermis in both humans and mice. The mRNA of *Hephl1*, but not *Heph* mRNA, is enriched in the uppermost layer of the mouse epidermis [60], and the HEPHL1 protein, but not HEPH, is elevated in the outer epidermis of human skin, as determined by quantitative proteomics [61]. Interestingly, HEPHL1 was detected as a component of mature hair shafts in mice [62] and humans [63], as determined by proteomic analysis. Furthermore, *HEPHL1* mRNA was detected in human pilosebaceous units, consisting of hair follicles and sebaceous glands, whereby its levels were significantly reduced in samples from patients with alopecia [64].

The function of HEPHL1 was tested by the targeted deletion of the corresponding gene in mice [65]. *Hephl1*-deficient mice are viable and develop a skin-specific phenotype, that is, the formation of curly whiskers [65]. Abnormal hair phenotypes are also associated with mutations of HEPHL1 in cw mice [66], cattle [67], and humans (pili torti and trichorrhexis nodosa) [65]. As the shape and structure of hair is determined by keratinocytes, these data support a role of HEPHL1 in epithelial cells of the skin. The investigation of fibroblasts from patients with *HEPHL1* loss-of-function mutations showed a significant increase in the intracellular iron content [65]. It will be interesting to determine the potential effects of *HEPHL1* mutations on the transport of iron in the skin and on the homeostasis of the epidermis.

## 4. Roles of Iron in Skin Infections and Other Pathologies

### 4.1. Control of Microbes on the Skin Surface

The surface of the skin is colonized by microbes which utilize nutrients, including iron, provided by the host for their own growth. The role of iron in the growth of microbes of pathological relevance for the skin and internal stratified epithelia, namely *S. aureus* [68,69,70] and *Candida albicans* [71], has been studied, but iron is essential for all microbes. Therefore, we discuss the possible role of iron in the control of the skin microbiome more broadly.

The skin microbiota is composed of a wide variety of bacteria, fungi, and viruses. The composition of the skin microbiome depends on moisture, amount of sebum, body site, and genetic factors that have not yet been fully defined [72,73,74]. Commonly, commensal and pathogenic microbes are distinguished, but usually harmless bacteria can cause damage under specific conditions [72]. Importantly, the microbiome is regulated by the composition of corneocytes and inter-corneocyte material (mainly lipids), the rate of shedding of corneocytes (desquamation), and soluble factors with anti-microbial activities [75,76]. The complex interplay of multiple factors maintains the homeostasis at the skin surface to limit microbial overgrowth and to suppress infections of the living layers of the skin. Upon access of microbes to the deeper layers of the epidermis or dermis, resident skin cells produce alarmins such as IL-1 and antimicrobial peptides. Professional immune cells contribute to the clearance of infectious microbes in the skin, as has been summarized previously in excellent reviews [77,78]. Metabolic adaptations of both host cells and microbes play a critical role under normal conditions of equilibrium and during dynamic changes in the skin surface, such as infections and wound healing. In the context of iron metabolism, it is essential to consider the dependence on iron of both host and microbes. Although a decrease in iron concentrations may limit the growth of microbes, it may even more strongly impair the anti-microbial defense mechanisms of the host.

Iron is an essential element for commensal and pathogenic bacteria and fungi [79,80]. The microbiota on the skin surface depends on the supply of iron from the epidermis whereby both free iron ions and heme are utilized by bacteria growing on the skin surface [79,80]. *Staphylococcus aureus*, which is abundant on the skin surface of patients with atopic dermatitis, prefers heme as a source of iron and depends on heme for efficient growth [68]. Accordingly, it could be hypothesized that the HO-1-dependent degradation of heme in the granular layer leads to reduced concentrations of heme on the skin surface and thereby limits the growth of *S. aureus* on the skin surface. Additionally, other skin bacteria, such as Corynebacterium [81] and Streptococcus [82], are likely to be differentially affected by changes in heme and iron levels. As *S. aureus* is able to enter living keratinocytes [83], the effects of iron on the intracellular growth of *S. aureus* are important but not well characterized.

### 4.2. Roles of Iron in Nutritional Immunity of the Skin

Nutritional immunity is based on the concept that the growth of microbial pathogens can be limited by host factors that sequester iron and other essential transition metals [70]. At the core of this defense strategy, the host competes with the microbe for nutrients. With regard to iron, the competition involves iron-binding proteins and the physical separation of domains with high and low iron concentrations.

Microbes have various iron acquisition systems, including the siderophores, receptors that bind mammalian iron-binding proteins and channels that import free Fe^2+^. Siderophores are secreted by the bacteria, bind free iron and subsequently support the internalization of iron by binding to receptors on the bacterial surface. Some bacteria utilize specific receptors for mammalian iron-binding molecules such as heme, transferrin or lactoferrin to obtain iron directly from these iron sources. The third mechanism of iron acquisition is the uptake of free Fe^2+^ via the transporter FeoB [69,84].

When iron is taken up by the bacteria, it binds to Fur (ferric uptake regulator), an iron-binding transcriptional regulator of a range of genes. Fur can serve as a transcriptional activator or repressor, with genes that are repressed by Fur being more sensitive to this regulation system in *E. coli* [85]. Fur has been shown to be important for bacterial growth as well as virulence factors and immunomodulatory molecules [86,87]. As a proposed virulence factor regulated by Fur, extracellular adherence protein has been identified as a responsible protein for delayed wound healing in *S. aureus*-infected wounds [88]. Another virulence factor identified to be Fur-dependent is coagulase [87]. Interestingly, coagulase was found to be produced only by wound- and burn-associated *S. aureus*, and not by *S. aureus* in the normal human microflora [89]. Furthermore, staphylococcal superantigen-like proteins Ssl11, 1, and 2 were found to be regulated by Fur [87]. Ssl virulence factors have been shown to play a role in host immune system regulation and are upregulated in the early stage of infection model [90]. Taken together, iron is an important contributor to the maintenance of the skin’s bacterial balance.

Epidermal keratinocytes secrete iron-binding proteins that limit the availability of iron and the iron-dependent growth of bacteria. Most notably, calprotectin is a protein complex that exists as either a heterodimer or heterotetramer of S100A8 and S100A9 proteins. Calprotectin can be produced by epidermal keratinocytes [91], but its abundance is very low under normal conditions, whereas psoriatic lesions [92] and UV-stressed epidermis are rich in S100A8 and S100A9 [93]. Calprotectin is a calcium-binding protein, but it can also chelate iron in the presence of calcium. Furthermore, it has been shown that iron chelation by calprotectin can minimize iron uptake from both Gram-positive and Gram-negative bacteria [94,95]. Notably, the utilization of heme is not blocked by calprotectin [96,97].

An additional mechanism by which the host limits the growth of microorganisms at the skin surface is the regulation of the total available iron amount in the outermost compartment of the epidermis. As described above, the export of iron from the keratinocytes in the upper granular layer of the epidermis allows the recycling of iron [9] and reduces the availability of iron to microorganisms on the skin surface.

### 4.3. Iron in Non-Infectious Skin Diseases

As this review cannot cover all aspects of the cutaneous iron metabolism in detail, we will mention only a few pathophysiologically relevant roles of iron which are clinically relevant and/or represent topics of current research. Iron-overload-associated conditions, such as hemochromatosis and chronic venous disease with increased extravasation of erythrocytes, are long-known clinical problems that affect the skin. An excess of iron is deposited in the skin in the form of hemosiderin, resulting in macroscopically visible brownish hyperpigmentation [98,99]. Hemosiderin granules are located extracellularly between the collagen bundles in the dermis and in the dermal macrophages, as well as in the epidermis, mostly in the Langerhans cells. In addition to direct hyperpigmentation by hemosiderin, melanogenesis was reported to be enhanced [98].

Iron is required for the stabilization of connective tissues because it serves as a cofactor, besides vitamin C, for lysyl hydroxylases, which catalyze the formation of hydroxylysine in procollagen [100]. Interestingly, a disturbance of the intracellular iron metabolism is hypothesized to impair lysyl hydroxylase activity and to cause a subtype of Ehlers–Danlos syndrome [101]. Additionally, the etiology of chronic wounds is critically regulated by iron and iron-dependent reactive oxygen species in fibroblasts and macrophages [102]. Furthermore, ferroptosis of various cell types is implicated in several skin diseases, such as vitiligo [103], melanoma [104] and rosacea [105], but whether it is indeed critical for the progression or control of these diseases remains to be tested.

## 5. Comparison of Iron Metabolism in the Epidermis and Other Stratified Epithelia

The epidermis of the skin is connected to other stratified epithelia that cover the oral cavity, the esophagus, and the vagina. These epithelia are not covered by a cornified layer in humans, but several differentiation steps are similar to those of the epidermis [106,107]. This similarity includes the expression of *Hmox1*, which was reported in the uppermost layers of the tongue, esophagus, and forestomach [54]. Unlike normal epidermis, oral epithelia constitutively produce the iron-binding antimicrobial protein calprotectin [108].

The effect of iron on infectious diseases of the mouth is complex because iron levels affect both microbes and immune defenses against microbes. For example, patients with iron deficiency were reported to have an elevated prevalence of oral candidosis [109]. Whether iron depletion by host factors critically controls oral microbes is not fully known [110]. Candida yeasts are killed by the iron-binding protein lactoferrin [111]. However, both the deprivation of iron and iron-independent interactions contribute to the antimicrobial activity of lactoferrin [112].

Interestingly, one subtype of oral epithelial cells, namely the ameloblasts, have evolved a most extreme adaptation of iron metabolism and transport. Ameloblasts on the labial side of rodent incisors secrete large amounts of iron into the forming enamel [113,114], so that the superficial layer of the incisors contains more than 5% iron [115]. This high iron content increases the hardness of the teeth [116]. The precise mechanism of iron transport through the ameloblasts of rodents is not known, but uniquely high levels of ferritin in the ameloblasts of mice point to a conservation of at least some key regulators of iron metabolism previously identified in other cell types [117]. The secretion of iron is blocked by the deletion of the *Nfe2l2* (Nrf2) gene [115] and by the deletion of the autophagy genes *Atg5* and *Atg7* in K14-positive epithelial cells and their progeny, including ameloblasts [118]. However, mutations of other genes that control ameloblast differentiation upstream of the pigmentation stage of enamel formation also impair the secretion of iron into the enamel. Recently, leukemia inhibitory factor, an interleukin 6 family cytokine, was reported to be required for the normal expression of TFR1 and ferroportin in ameloblasts and for the secretion of iron [119].

## 6. Conclusions and Perspectives

Based on the currently available literature on iron in the skin, as summarized above, we conclude this review by highlighting some key points and directions for future research. Accumulating evidence suggests that the epidermis of the skin plays an active role in the control of systemic iron balance. Moreover, the turnover of iron within the epidermis is subjected to tight regulation. In particular, epidermal regeneration and differentiation are linked to the utilization of iron in multiple biochemical reactions, as well as the recycling, transport, and release of iron. The passage of iron to the surface of the epidermis supplies microbes with iron, and thereby affects the microbiome composition in healthy and diseased skin. While some basic features of iron turnover are conserved between the epidermis and stratified epithelia of the oral cavity, the enamel epithelium of rodents has evolved a unique modification of epithelial iron transport that facilitates a high rate of iron secretion. Mutations of *HEPHL1* suggest that hair follicles have particular requirements with regard to iron turnover.

At present, there are many important gaps in the knowledge of cutaneous iron metabolism. The concept of the partial recycling of iron in the epidermis [9] requires more direct evidence for the flow of iron through the layers of the epidermis and the link between epidermal and systemic iron levels [120]. It will be interesting to determine which hemoproteins play critical roles in differentiating keratinocytes and by which mechanisms they are degraded to release heme, the substrate of heme oxygenase 1 in the granular layer. The mechanisms of the transcriptional upregulation of *HMOX1* and *SLC40A1* in the upper layers of the epidermis and possible aberrations thereof in diseased skin need to be investigated. The link between the excretion of iron and the skin microbiome has not been well characterized thus far. It remains to be tested whether iron or other trace elements play critical roles in the nutritional immunity of the skin. The control of iron metabolism in skin appendages, such as hair and sweat glands, with potential implications for their regenerative potential, should be investigated in more depth. With regard to the methodology of research of iron metabolism, studies in both transgenic mouse models and human tissue samples and cells remain important. The use of advanced methods of imaging Fe^2+^ [121] and gene expression analysis, such as single-cell RNA sequencing and spatial mass-spectrometry-based proteomics [122], will allow the determination of the organization of iron turnover with high resolution.

## References

[1] Fuchs E. (2007). Scratching the surface of skin development. Nature.

[2] Watt F.M. (1989). Terminal differentiation of epidermal keratinocytes. Curr. Opin. Cell Biol..

[3] Matsui T., Amagai M. (2015). Dissecting the formation, structure and barrier function of the stratum corneum. Int. Immunol..

[4] Eckhart L., Zeeuwen P.L.J.M. (2018). The skin barrier: Epidermis vs environment. Exp. Dermatol..

[5] Dutt S., Hamza I., Bartnikas T.B. (2022). Molecular mechanisms of iron and heme metabolism. Annu. Rev. Nutr..

[6] Ricci A., Di Betto G., Bergamini E., Buzzetti E., Corradini E., Ventura P. (2022). Iron metabolism in the disorders of heme biosynthesis. Metabolites.

[7] Rizzollo F., More S., Vangheluwe P., Agostinis P. (2021). The lysosome as a master regulator of iron metabolism. Trends. Biochem. Sci..

[8] Wright J.A., Richards T., Srai S.K. (2014). The role of iron in the skin and cutaneous wound healing. Front. Pharmacol..

[9] Asano M., Yamasaki K., Yamauchi T., Terui T., Aiba S. (2017). Epidermal iron metabolism for iron salvage. J. Dermatol. Sci..

[10] Surbek M., Sukseree S., Sachslehner A.P., Copic D., Golabi B., Nagelreiter I.M., Tschachler E., Eckhart L. (2023). Heme oxygenase-1 is upregulated during differentiation of keratinocytes but its expression is dispensable for cornification of murine epidermis. J. Dev. Biol..

[11] Caldas Nogueira M.L., Pastore A.J., Davidson V.L. (2021). Diversity of structures and functions of oxo-bridged non-heme diiron proteins. Arch. Biochem. Biophys..

[12] Kotla N.K., Dutta P., Parimi S., Das N.K. (2022). The role of ferritin in health and disease: Recent advances and understandings. Metabolites.

[13] De Domenico I., McVey Ward D., Kaplan J. (2008). Regulation of iron acquisition and storage: Consequences for iron-linked disorders. Nat. Rev. Mol. Cell Biol..

[14] Chen X., Yu C., Kang R., Tang D. (2020). Iron metabolism in ferroptosis. Front. Cell Dev. Biol..

[15] Vats K., Kruglov O., Mizes A., Samovich S.N., Amoscato A.A., Tyurin V.A., Tyurina Y.Y., Kagan V.E., Bunimovich Y.L. (2021). Keratinocyte death by ferroptosis initiates skin inflammation after UVB exposure. Redox Biol..

[16] Liu L., Lian N., Shi L., Hao Z., Chen K. (2023). Ferroptosis: Mechanism and connections with cutaneous diseases. Front. Cell Dev. Biol..

[17] Shah R., Shchepinov M.S., Pratt D.A. (2018). Resolving the role of lipoxygenases in the initiation and execution of ferroptosis. ACS Cent. Sci..

[18] Shou Y., Yang L., Yang Y., Xu J. (2021). Inhibition of keratinocyte ferroptosis suppresses psoriatic inflammation. Cell Death Dis..

[19] Krieg P., Fürstenberger G. (2014). The role of lipoxygenases in epidermis. Biochim. Biophys. Acta.

[20] Egolf S., Zou J., Anderson A., Simpson C.L., Aubert Y., Prouty S., Ge K., Seykora J.T., Capell B.C. (2021). MLL4 mediates differentiation and tumor suppression through ferroptosis. Sci. Adv..

[21] Jobard F., Lefèvre C., Karaduman A., Blanchet-Bardon C., Emre S., Weissenbach J., Ozgüc M., Lathrop M., Prud’homme J.F., Fischer J. (2002). Lipoxygenase-3 (ALOXE3) and 12(R)-lipoxygenase (ALOX12B) are mutated in non-bullous congenital ichthyosiform erythroderma (NCIE) linked to chromosome 17p13.1. Hum. Mol. Genet..

[22] Youssry I., Mohsen N.A., Shaker O.G., El-Hennawy A., Fawzy R., Abu-Zeid N.M., El-Beshlawy A. (2007). Skin iron concentration: A simple, highly sensitive method for iron stores evaluation in thalassemia patients. Hemoglobin.

[23] Khalil S., Cavagnero K.J., Williams M.R., O’Neill A., Nakatsuji T., Gallo R.L. (2023). Regulation of epidermal ferritin expression influences systemic iron homeostasis. J. Investig. Dermatol..

[24] Weintraub L.R., Demis D.J., Conrad M.E., Crosby W.H. (1965). Iron excretion by the skin. Selective localization or iron-59 in epthelial cells. Am. J. Pathol..

[25] Milstone L.M., Hu R.H., Dziura J.D., Zhou J. (2012). Impact of epidermal desquamation on tissue stores of iron. J. Dermatol. Sci..

[26] Christophers E., Sterry W., Fitzpatrick T.B., Eisen A.Z., Wolff K., Freedberg I.M., Austen K.F. (1993). Epidermis: Disorders of cell kinetics and differentiation. Dermatology in General Medicine.

[27] Ponikowska M., Tupikowska M., Kasztura M., Jankowska E.A., Szepietowski J.C. (2015). Deranged iron status in psoriasis: The impact of low body mass. J. Cachexia Sarcopenia Muscle.

[28] Coger V., Million N., Rehbock C., Sures B., Nachev M., Barcikowski S., Wistuba N., Strauß S., Vogt P.M. (2019). Tissue concentrations of zinc, iron, copper, and magnesium during the phases of full thickness wound healing in a rodent model. Biol. Trace Elem. Res..

[29] Molin L., Wester P.O. (1973). Iron content in normal and psoriatic epidermis. Acta Derm. Venereol..

[30] Forslind B., Werner-Linde Y., Lindberg M., Pallon J. (1999). Elemental analysis mirrors epidermal differentiation. Acta Derm. Venereol..

[31] Soyer H.P., Smolle J., Torne R., Kerl H. (1987). Transferrin receptor expression in normal skin and in various cutaneous tumors. J. Cutan. Pathol..

[32] Metral E., Bechetoille N., Demarne F., Rachidi W., Damour O. (2017). α6 Integrin (α6high)/Transferrin receptor (CD71)low keratinocyte stem cells are more potent for generating reconstructed skin epidermis than rapid adherent cells. Int. J. Mol. Sci..

[33] Milstone L.M., Adams B.D., Zhou J., Bruegel Sanchez V.L., Shofner J. (2006). Stratum-specific expression of human transferrin receptor increases iron in mouse epidermis. J. Investig. Dermatol..

[34] Applegate L.A., Scaletta C., Panizzon R., Frenk E. (1998). Evidence that ferritin is UV inducible in human skin: Part of a putative defense mechanism. J. Investig. Dermatol..

[35] Applegate L.A., Frenk E. (1995). Oxidative defense in cultured human skin fibroblasts and keratinocytes from sun-exposed and non-exposed skin. Photodermatol. Photoimmunol. Photomed..

[36] Kerins M.J., Ooi A. (2018). The roles of NRF2 in modulating cellular iron homeostasis. Antioxid. Redox Signal..

[37] Yanatori I., Nishina S., Kishi F., Hino K. (2023). Newly uncovered biochemical and functional aspects of ferritin. FASEB J..

[38] Mancias J.D., Wang X., Gygi S.P., Harper J.W., Kimmelman A.C. (2014). Quantitative proteomics identifies NCOA4 as the cargo receptor mediating ferritinophagy. Nature.

[39] Seité S., Popovic E., Verdier M.P., Roguet R., Portes P., Cohen C., Fourtanier A., Galey J.B. (2004). Iron chelation can modulate UVA-induced lipid peroxidation and ferritin expression in human reconstructed epidermis. Photodermatol. Photoimmunol. Photomed..

[40] Hasegawa S., Harada K., Morokoshi Y., Tsukamoto S., Furukawa T., Saga T. (2013). Growth retardation and hair loss in transgenic mice overexpressing human H-ferritin gene. Transgen. Res..

[41] Anderson C.P., Shen M., Eisenstein R.S., Leibold E.A. (2012). Mammalian iron metabolism and its control by iron regulatory proteins. Biochim. Biophys. Acta..

[42] Giordani A., Martin M.E., Beaumont C., Santus R., Morlière P. (2000). Inactivation of iron responsive element-binding capacity and aconitase function of iron regulatory protein-1 of skin cells by ultraviolet A. Photochem. Photobiol..

[43] Malerba M., Louis S., Cuvellier S., Shambat S.M., Hua C., Gomart C., Fouet A., Ortonne N., Decousser J.W., Zinkernagel A.S. (2020). Epidermal hepcidin is required for neutrophil response to bacterial infection. J. Clin. Investig..

[44] Cavill I., Jacobs A., Beamish M., Owen G. (1969). Iron turnover in the skin. Nature.

[45] Eckhart L., Lippens S., Tschachler E., Declercq W. (2013). Cell death by cornification. Biochim. Biophys. Acta..

[46] Yokouchi M., Kubo A. (2018). Maintenance of tight junction barrier integrity in cell turnover and skin diseases. Exp. Dermatol..

[47] De Benedetto A., Rafaels N.M., McGirt L.Y., Ivanov A.I., Georas S.N., Cheadle C., Berger A.E., Zhang K., Vidyasagar S., Yoshida T. (2011). Tight junction defects in patients with atopic dermatitis. J. Allergy Clin. Immunol..

[48] Kirschner N., Houdek P., Fromm M., Moll I., Brandner J.M. (2010). Tight junctions form a barrier in human epidermis. Eur. J. Cell Biol..

[49] Méhul B., Ménigot C., Fogel P., Seraidaris A., Genette A., Pascual T., Duvic M., Voegel J.J. (2019). Proteomic analysis of stratum corneum in cutaneous T-cell lymphomas and psoriasis. Exp. Dermatol..

[50] Campbell N.K., Fitzgerald H.K., Dunne A. (2021). Regulation of inflammation by the antioxidant haem oxygenase 1. Nat. Rev. Immunol..

[51] Wojas-Pelc A., Marcinkiewicz J. (2007). What is a role of haeme oxygenase-1 in psoriasis? Current concepts of pathogenesis. Int. J. Exp. Pathol..

[52] Xiang Y., Liu G., Yang L., Zhong J.L. (2011). UVA-induced protection of skin through the induction of heme oxygenase-1. Biosci. Trends.

[53] Numata I., Okuyama R., Memezawa A., Ito Y., Takeda K., Furuyama K., Shibahara S., Aiba S. (2009). Functional expression of heme oxygenase-1 in human differentiated epidermis and its regulation by cytokines. J. Investig. Dermatol..

[54] McMahon M., Ding S., Acosta-Jimenez L.P., Frangova T.G., Henderson C.J., Wolf C.R. (2018). Measuring in vivo responses to endogenous and exogenous oxidative stress using a novel haem oxygenase 1 reporter mouse. J. Physiol..

[55] Udayanga K.G., Miyata H., Yokoo Y., Qi W.M., Takahara E., Mantani Y., Yokoyama T., Hoshi N., Kitagawa H. (2011). Immunohistochemical study of the apoptosis process in epidermal epithelial cells of rats under a physiological condition. Histol. Histopathol..

[56] Schäfer M., Farwanah H., Willrodt A.H., Huebner A.J., Sandhoff K., Roop D., Hohl D., Bloch W., Werner S. (2012). Nrf2 links epidermal barrier function with antioxidant defense. EMBO Mol. Med..

[57] Ishitsuka Y., Roop D.R. (2021). The epidermis: Redox governor of health and diseases. Antioxidants.

[58] Yang Q., Liu W., Zhang S., Liu S. (2020). The cardinal roles of ferroportin and its partners in controlling cellular iron in and out. Life Sci..

[59] Vashchenko G., MacGillivray R.T. (2013). Multi-copper oxidases and human iron metabolism. Nutrients.

[60] Matsui T., Kadono-Maekubo N., Suzuki Y., Furuichi Y., Shiraga K., Sasaki H., Ishida A., Takahashi S., Okada T., Toyooka K. (2021). A unique mode of keratinocyte death requires intracellular acidification. Proc. Natl. Acad. Sci. USA.

[61] Dyring-Andersen B., Løvendorf M.B., Coscia F., Santos A., Møller L.B.P., Colaço A.R., Niu L., Bzorek M., Doll S., Andersen J.L. (2020). Spatially and cell-type resolved quantitative proteomic atlas of healthy human skin. Nat. Commun..

[62] Sukseree S., Karim N., Jaeger K., Zhong S., Rossiter H., Nagelreiter I.M., Gruber F., Tschachler E., Rice R.H., Eckhart L. (2023). Autophagy controls the protein composition of hair shafts. J. Investig. Dermatol..

[63] Laatsch C.N., Durbin-Johnson B.P., Rocke D.M., Mukwana S., Newland A.B., Flagler M.J., Davis M.G., Eigenheer R.A., Phinney B.S., Rice R.H. (2014). Human hair shaft proteomic profiling: Individual differences, site specificity and cuticle analysis. PeerJ.

[64] Samra E.B., Mahé Y.F., Le Balch M., Cavusoglu N., Bouhanna P., Bakkar K. (2021). Transcriptome profiling of pilosebaceous units in male androgenetic alopecia reveals altered junctional networks. J. Investig. Dermatol..

[65] Sharma P., Reichert M., Lu Y., Markello T.C., Adams D.R., Steinbach P.J., Fuqua B.K., Parisi X., Kaler S.G., Vulpe C.D. (2019). Biallelic HEPHL1 variants impair ferroxidase activity and cause an abnormal hair phenotype. PLoS Genet..

[66] Eragene S., Stewart J.J., Samuel-Constanzo J.I., Tan T., Esgdaille N.Z., Bigiarelli K.J., DaCosta V.D., Jimenez H., King T.R. (2019). The mouse curly whiskers (cw) mutations are recessive alleles of hephaestin-like 1 (Hephl1). Mol. Genet. Metab. Rep..

[67] Kuca T., Marron B.M., Jacinto J.G.P., Paris J.M., Gerspach C., Beever J.E., Drögemüller C. (2021). A Nonsense variant in hephaestin like 1 (HEPHL1) is responsible for congenital hypotrichosis in belted Galloway cattle. Genes.

[68] Skaar E.P., Humayun M., Bae T., DeBord K.L., Schneewind O. (2004). Iron-source preference of *Staphylococcus aureus* infections. Science.

[69] van Dijk M.C., de Kruijff R.M., Hagedoorn P.L. (2022). The role of iron in *Staphylococcus aureus* infection and human disease: A metal tug of war at the host-microbe interface. Front. Cell Dev. Biol..

[70] Murdoch C.C., Skaar E.P. (2022). Nutritional immunity: The battle for nutrient metals at the host-pathogen interface. Nat. Rev. Microbiol..

[71] Fourie R., Kuloyo O.O., Mochochoko B.M., Albertyn J., Pohl C.H. (2018). Iron at the centre of Candida albicans interactions. Front. Cell. Infect. Microbiol..

[72] Byrd A., Belkaid Y., Segre J. (2018). The human skin microbiome. Nat. Rev. Microbiol..

[73] Chen Y., Fischbach M., Belkaid Y. (2018). Skin microbiota–host interactions. Nature.

[74] Ederveen T.H.A., Smits J.P.H., Boekhorst J., Schalkwijk J., van den Bogaard E.H., Zeeuwen P.L.J.M. (2020). Skin microbiota in health and disease: From sequencing to biology. J. Dermatol..

[75] Schröder J.M. (2010). The role of keratinocytes in defense against infection. Curr. Opin. Infect. Dis..

[76] Rademacher F., Gläser R., Harder J. (2021). Antimicrobial peptides and proteins: Interaction with the skin microbiota. Exp. Dermatol..

[77] Kabashima K., Honda T., Ginhoux F., Egawa G. (2019). The immunological anatomy of the skin. Nat. Rev. Immunol..

[78] Kobayashi T., Naik S., Nagao K. (2019). Choreographing immunity in the skin epithelial barrier. Immunity.

[79] Haley K.P., Janson E.M., Heilbronner S., Foster T.J., Skaar E.P. (2011). Staphylococcus lugdunensis IsdG liberates iron from host heme. J. Bacteriol..

[80] Lyles K.V., Eichenbaum Z. (2018). From host heme to iron: The expanding spectrum of heme degrading enzymes used by pathogenic bacteria. Front. Cell. Infect. Microbiol..

[81] Ibraim I.C., Parise M.T.D., Parise D., Sfeir M.Z.T., de Paula Castro T.L., Wattam A.R., Ghosh P., Barh D., Souza E.M., Góes-Neto A. (2019). Transcriptome profile of Corynebacterium pseudotuberculosis in response to iron limitation. BMC Genom..

[82] Ge R., Sun X. (2014). Iron acquisition and regulation systems in Streptococcus species. Metallomics.

[83] Kintarak S., Whawell S.A., Speight P.M., Packer S., Nair S.P. (2004). Internalization of *Staphylococcus aureus* by human keratinocytes. Infect. Immun..

[84] Schaible U.E., Kaufmann S.H. (2004). Iron and microbial infection. Nat. Rev. Microbiol..

[85] Hou C., Liu L., Ju X., Xiao Y., Li B., You C. (2023). Revisiting Fur Regulon Leads to a Comprehensive Understanding of Iron and Fur Regulation. Int. J. Mol. Sci..

[86] Horsburgh M.J., Ingham E., Foster S.J. (2001). In *Staphylococcus aureus*, fur is an interactive regulator with PerR, contributes to virulence, and is necessary for oxidative stress resistance through positive regulation of catalase and iron homeostasis. J. Bacteriol..

[87] Torres V.J., Attia A.S., Mason W.J., Hood M.I., Corbin B.D., Beasley F.C., Anderson K.L., Stauff D.L., McDonald W.H., Zimmerman L.J. (2010). *Staphylococcus aureus* fur regulates the expression of virulence factors that contribute to the pathogenesis of pneumonia. Infect. Immun..

[88] Athanasopoulos A.N., Economopoulou M., Orlova V.V., Sobke A., Schneider D., Weber H., Augustin H.G., Eming S.A., Schubert U., Linn T. (2006). The extracellular adherence protein (Eap) of *Staphylococcus aureus* inhibits wound healing by interfering with host defense and repair mechanisms. Blood.

[89] Abdulbaqi A., Ibrahim A.S. (2023). Molecular analysis of *Staphylococcus aureus* isolated from clinical samples and natural flora. Cell. Mol. Biol. (Noisy-le-Grand).

[90] Bretl D.J., Elfessi A., Watkins H., Schwan W.R. (2019). Regulation of the *Staphylococcal* superantigen-like protein 1 gene of community-associated methicillin-resistant *Staphylococcus aureus* in murine abscesses. Toxins.

[91] Broome A.M., Ryan D., Eckert R.L. (2003). S100 protein subcellular localization during epidermal differentiation and psoriasis. J. Histochem. Cytochem..

[92] Matsunaga Y., Hashimoto Y., Ishiko A. (2021). Stratum corneum levels of calprotectin proteins S100A8/A9 correlate with disease activity in psoriasis patients. J. Dermatol..

[93] Grimbaldeston M.A., Geczy C.L., Tedla N., Finlay-Jones J.J., Hart P.H. (2003). S100A8 induction in keratinocytes by ultraviolet A irradiation is dependent on reactive oxygen intermediates. J. Investig. Dermatol..

[94] Nakashige T.G., Zhang B., Krebs C., Nolan E.M. (2015). Human calprotectin is an iron-sequestering host-defense protein. Nat. Chem. Biol..

[95] Obisesan A.O., Zygiel E.M., Nolan E.M. (2021). Bacterial responses to iron withholding by calprotectin. Biochemistry.

[96] Zygiel E.M., Nolan E.M. (2018). Transition metal sequestration by the host-defense protein calprotectin. Annu. Rev. Biochem..

[97] Zygiel E.M., Obisesan A.O., Nelson C.E., Oglesby A.G., Nolan E.M. (2021). Heme protects Pseudomonas aeruginosa and *Staphylococcus aureus* from calprotectin-induced iron starvation. J. Biol. Chem..

[98] Tsuji T. (1980). Experimental hemosiderosis: Relationship between skin pigmentation and hemosiderin. Acta Derm. Venereol..

[99] Caggiati A., Rosi C., Casini A., Cirenza M., Petrozza V., Acconcia M.C., Zamboni P. (2010). Skin iron deposition characterises lipodermatosclerosis and leg ulcer. Eur. J. Vasc. Endovasc. Surg..

[100] Pinnell S.R., Krane S.M., Kenzora J.E., Glimcher M.J. (1972). A heritable disorder of connective tissue. Hydroxylysine-deficient collagen disease. N. Engl. J. Med..

[101] Xiao G., Zhou B. (2018). ZIP13: A study of Drosophila offers an alternative explanation for the corresponding human disease. Front. Genet..

[102] Wlaschek M., Singh K., Sindrilaru A., Crisan D., Scharffetter-Kochanek K. (2019). Iron and iron-dependent reactive oxygen species in the regulation of macrophages and fibroblasts in non-healing chronic wounds. Free Radic. Biol. Med..

[103] Wu X., Jin S., Yang Y., Lu X., Dai X., Xu Z., Zhang C., Xiang L.F. (2022). Altered expression of ferroptosis markers and iron metabolism reveals a potential role of ferroptosis in vitiligo. Pigment Cell Melanoma Res..

[104] Wang S., Yi X., Wu Z., Guo S., Dai W., Wang H., Shi Q., Zeng K., Guo W., Li C. (2022). CAMKK2 defines ferroptosis sensitivity of melanoma cells by regulating AMPK–NRF2 pathway. J. Investig. Dermatol..

[105] Hu X.M., Zheng S.Y., Mao R., Zhang Q., Wan X.X., Zhang Y.Y., Li J., Yang R.H., Xiong K. (2023). Pyroptosis-related gene signature elicits immune response in rosacea. Exp. Dermatol..

[106] Wagner T., Beer L., Gschwandtner M., Eckhart L., Kalinina P., Laggner M., Ellinger A., Gruber R., Kuchler U., Golabi B. (2019). The differentiation-associated keratinocyte protein cornifelin contributes to cell-cell adhesion of epidermal and mucosal keratinocytes. J. Investig. Dermatol..

[107] Sachslehner A.P., Surbek M., Golabi B., Geiselhofer M., Jäger K., Hess C., Kuchler U., Gruber R., Eckhart L. (2023). Transglutaminase activity is conserved in stratified epithelia and skin appendages of mammals and birds. Int. J. Mol. Sci..

[108] Argyris P.P., Slama Z.M., Ross K.F., Khammanivong A., Herzberg M.C. (2018). Calprotectin and the initiation and progression of head and neck cancer. J. Dent. Res..

[109] Lu S.Y. (2016). Perception of iron deficiency from oral mucosa alterations that show a high prevalence of Candida infection. J. Formos. Med. Assoc..

[110] Solis N.V., Wakade R.S., Filler S.G., Krysan D.J. (2023). Candida albicans oropharyngeal infection is an exception to iron-based nutritional immunity. mBio.

[111] Nikawa H., Samaranayake L.P., Tenovuo J., Pang K.M., Hamada T. (1993). The fungicidal effect of human lactoferrin on Candida albicans and Candida krusei. Arch. Oral. Biol..

[112] Gruden Š., Poklar Ulrih N. (2021). Diverse mechanisms of antimicrobial activities of lactoferrins, lactoferricins, and other lactoferrin-derived peptides. Int. J. Mol. Sci..

[113] Muto T., Miyoshi K., Horiguchi T., Noma T. (2012). Dissection of morphological and metabolic differentiation of ameloblasts via ectopic SP6 expression. J. Med. Investig..

[114] Wen X., Paine M.L. (2013). Iron deposition and ferritin heavy chain (Fth) localization in rodent teeth. BMC Res. Notes.

[115] Yanagawa T., Itoh K., Uwayama J., Shibata Y., Yamaguchi A., Sano T., Ishii T., Yoshida H., Yamamoto M. (2004). Nrf2 deficiency causes tooth decolourization due to iron transport disorder in enamel organ. Genes Cells.

[116] Gordon L.M., Cohen M.J., MacRenaris K.W., Pasteris J.D., Seda T., Joester D. (2015). Dental materials. Amorphous intergranular phases control the properties of rodent tooth enamel. Science.

[117] Miyazaki Y., Sakai H., Shibata Y., Shibata M., Mataki S., Kato Y. (1998). Expression and localization of ferritin mRNA in ameloblasts of rat incisor. Arch. Oral. Biol..

[118] Sukseree S., Schwarze U.Y., Gruber R., Gruber F., Quiles Del Rey M., Mancias J.D., Bartlett J.D., Tschachler E., Eckhart L. (2020). ATG7 is essential for secretion of iron from ameloblasts and normal growth of murine incisors during aging. Autophagy.

[119] Fan L., Ou Y.J., Zhu Y.X., Liang Y.D., Zhou Y., Wang Y.N. (2022). Lif deficiency leads to iron transportation dysfunction in ameloblasts. J. Dent. Res..

[120] Poss K.D., Tonegawa S. (1997). Heme oxygenase 1 is required for mammalian iron reutilization. Proc. Natl. Acad. Sci. USA.

[121] Hirayama T. (2019). Fluorescent probes for the detection of catalytic Fe(II) ion. Free Radic. Biol. Med..

[122] Mund A., Brunner A.D., Mann M. (2022). Unbiased spatial proteomics with single-cell resolution in tissues. Mol. Cell.

